# Down-regulation of THBS2 inhibits the malignant progression of Burkitt lymphoma by blocking the PI3K/AKT/c-MYC pathway

**DOI:** 10.3389/fonc.2026.1879902

**Published:** 2026-07-24

**Authors:** Yahong Xu, Shengquan Liu, Shaoxiong Wang, Yuanling Huang

**Affiliations:** Department of Hematology, First Hospital of Quanzhou Affiliated to Fujian Medical University, Quanzhou, Fujian, China

**Keywords:** apoptosis, Burkitt lymphoma, Cell Cycle, PI3K/AKT/c-MYC pathway, THBS2

## Abstract

**Background:**

Thrombospondin 2 (THBS2) is overexpressed in various solid tumors and is a prognostic gene of Burkitt lymphoma (BL), but its role and molecular mechanism in BL still need to be explored.

**Methods:**

Western blot (WB) detected the protein expression of THBS2 in BL cells. si-THBS2 was transfected into CA46 and RAJI cells to detect cell malignant phenotype and cell cycle changes. WB detected the protein expression of epithelial-mesenchymal transition (EMT), apoptosis, cell cycle and the PI3K/AKT/c-MYC pathway proteins. ChIP-qPCR detected the binding ability of MYC to target gene promoters, and Co-IP detected the level of MYC/MAX dimerization. Tumor stem cell sphere formation assays evaluated cell stemness changes. The subcutaneous xenograft model of RAJI cells was developed to observe the effect of THBS2 knockdown on tumor growth.

**Results:**

THBS2 protein was markedly up-regulated in BL cells. Knockdown of THBS2 could inhibit the growth and EMT process, inhibit the protein levels of cyclins (CDK2, CDK4, CDK6 and CyclinD1), inhibit the sphere formation ability of stem cells and down-regulate SOX2 and OCT4 expressions. THBS2 knockdown inhibited the PI3K/AKT pathway phosphorylation, declined c-MYC expressions, weakened the MYC/MAX dimerization and the binding ability of MYC to target gene promoters. In addition, THBS2 knockdown also significantly inhibited the growth of subcutaneous xenografts of RAJI cells, reduced the positive rates of Ki67 and PCNA, declined the protein levels of cyclins, and inhibited the PI3K/AKT/c-MYC pathway. Overexpression of c-MYC or 740Y-P treatment partially reversed the tumor malignant phenotype inhibition caused by THBS2 knockdown and partially restored cyclin levels.

**Conclusion:**

Knockdown of THBS2 inhibited the malignant phenotype, EMT and stem cell-like characteristics via inhibiting the PI3K/AKT/c-MYC signaling axis, and blocked the cell cycle.

## Graphical Abstract

**Figure:**
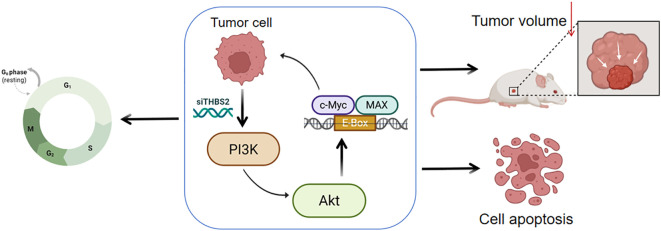
Knockdown of THBS2 inhibits the malignant progression and stem cell-like characteristics of BL cells through the PI3K/AKT/c-MYC signaling pathway, while promoting apoptosis and inducing cell cycle arrest.

## Highlights

- The expression of THBS2 is up-regulated in Burkitt lymphoma (BL).
- Down-regulation of THBS2 inhibits the malignant progression and stem cell-like characteristics of BL cells and promotes cell cycle arrest.
- Down-regulation of THBS2 inhibits the PI3K/AKT/c-MYC axis.
- Down-regulation of THBS2 suppresses tumor growth through the PI3K/AKT/c-MYC signaling pathway.

## 1 Introduction

Burkitt lymphoma (BL) is characterized by germinal center phenotype and almost universal MYC oncogene translocation to enhancer region. It has strong cell proliferation ability (proliferation index usually more than 95% and rapid clinical progression (1–3). Epidemiological data show that the disease accounts for about 40% of non-Hodgkin’s lymphoma in children in the USA, and there is a significant gender difference (male: female = 39: 11) (4). The most common primary site of the disease is the abdomen and lymphoid tissue (5). At present, the treatment strategy of BL is similar to that of diffuse large B-cell lymphoma, which mainly depends on chemotherapy regimens, such as DA-EPOCH-R regimen, high-dose methotrexate and cytarabine (6, 7). Although these treatments have achieved remarkable results, the serious side effects of chemotherapy cannot be ignored. Therefore, developing safer and more efficacious new treatments are urgently needed.

In recent years, targeted therapy strategies for BL molecular pathways have attracted much attention. The occurrence of BL lymphoma involves abnormal activation of multiple signaling pathways, including cell cycle disorders, apoptosis inhibition, and abnormal epigenetic regulation (8). Among them, phosphatidylinositol 3-kinase/protein kinase B (PI3K/AKT) pathway is involved in regulating various cellular functions, including apoptosis, migration and glucose transport, and is essential for maintaining cell homeostasis. The imbalance of this pathway is central to the pathogenesis of BL (9). Moreover, the pathogenesis of BL also involves c-MYC translocation and overexpression (10, 11). Overexpression of MYC activates PI3K, which in turn phosphorylates AKT, promotes cell survival, and inhibits apoptosis (12). AKT phosphorylation levels are markedly increased in BL cell lines, and PI3K inhibitors enhance BL cell sensitivity to chemotherapy (12, 13). Previous studies have shown that synergistic inhibition of survivin and the PI3K/AKT pathways can exert anti-lymphoma effects (14). Inhibition of MYC gene expression can inhibit the survival of BL cells (15). It has been reported that quercetin induces apoptosis of BL cells by synergistically reducing c-MYC expression and inhibiting the PI3K/AKT signaling pathway (10). These studies emphasize the important value of the PI3K/AKT/c-MYC signaling pathway as a therapeutic target for BL.

Thrombospondin-2 (THBS2) is a secreted trimeric extracellular matrix glycoprotein, which belongs to the thrombospondin family and participates in the regulation of extracellular matrix remodeling, angiogenesis and cell adhesion (16, 17). Recently, the role of THBS2 in tumors has received extensive attention, but its function is highly tissue-specific. In colorectal cancer, THBS2 is overexpressed in tumors and is related to advanced TNM staging and poor prognosis of patients (18). Epithelial-mesenchymal transition (EMT) is a key biological process for tumor cells to acquire the ability of invasion and metastasis. It is characterized by the down-regulation of epithelial marker E-cadherin and the up-regulation of mesenchymal markers N-cadherin and Vimentin, which promote cells to penetrate tissue barriers and enhance migration (19, 20). Mechanistic studies have shown that THBS2 can promote the EMT process by activating the PI3K/AKT signaling pathway (21). In lung adenocarcinoma, THBS2 can directly bind to the SDC4 receptor and enhance the EMT process (22). Furthermore, RNA-seq analysis suggested that THBS2 was a biomarker for the prognosis of BL, and enrichment analysis showed that it was associated with the PI3K/AKT axis (23). Moreover, it has been found that THBS2 activates this signaling in an INHBA-dependent manner, thereby promoting the malignant progression of prostate cancer (21). These studies indicate that THBS2 likely regulates the malignant biological behavior of BL via the PI3K/AKT/c-MYC axis.

Previous work has confirmed that inhibiting the PI3K/AKT axis activation can inhibit BL growth (24). The expression characteristics and biological functions of THBS2 in BL are still undefined. This study intends to detect THBS2 levels in BL cells, systematically explore the effect of THBS2 on tumor progression through functional experiments and transplanted tumor models, and clarify the molecular mechanism of THBS2 regulating the progression of BL, so as to provide new theoretical basis and potential intervention targets for targeted therapy of BL.

## 2 Methods

### 2.1 Construction of THBS2 stable overexpression BL cells

Human peripheral blood B lymphocytes IM-9 and human BL cells (CA46, NAMALWA, Daudi and RAJI) were purchased from SUNNCELL (SNL-637, SNL-133, SNL-046, SNL-039, SNL-048, Wuhan, China). The cells were cultured in RPMI 1640 complete medium (containing 10% fetal bovine serum and 1% penicillin-streptomycin, SNM-001E, SUNNCELL) at 37°C, 5% CO_2_ saturated humidity incubator (HERAcell 150i, Thermo Fisher, Waltham, MA, USA).

BL cells were transfected with si-THBS2, si-MYC and oe-MYC plasmids (Genesil, Wuhan, China) using Lipofectamine™ 3000 transfection reagent (L3000001, Invitrogen, Austin, TX, USA). One day before transfection, BL cells were seeded in 6-well plates (1×10^6^ cells/well). 30 min before transfection, the medium was replaced with fresh serum-free, antibiotic-free RPMI 1640 medium. 2 μg si-THBS2, si-MYC and oe-MYC plasmids were taken and added to 50 μL RPMI 1640 medium, gently mixed and recorded as solution A; 5 μL LipofectamineTM 3000 transfection reagent was added to 50 μL RPMI 1640 medium and stood for 5 min, which was recorded as B solution. The A and B solutions were mixed and incubated for 20 min to form a stable liposome complex with the transfection reagent. The liposome complex was then added to the cell culture medium and routinely cultured for 6 h. Subsequently, the medium was replaced with RPMI 1640 complete medium and cultured for 24 h. The protein levels were detected, and the relative expressions of THBS2 and MYC were calculated based on si-NC or oe-NC to verify the transfection efficiency.

Transfected BL cells were treated with 10 μM PI3K agonist 740Y-P (HY-P0175, MedChemExpress, Monmouth Junction, NJ, USA) and 10 μM PI3K inhibitor LY294002 (dissolved in dimethyl sulfoxide (DMSO), HY-10108, MedChemExpress) for 24 h.

### 2.2 Clone formation experiment

1.5 mL of RPMI 1640 complete medium containing 0.6% agarose was added and coagulated at room temperature. BL cells were resuspended in complete medium containing 0.3% agarose, and rapidly inoculated on the solidified bottom agar. After solidification at room temperature, the plates were placed for 10–21 days until cell colonies with a diameter greater than 75 μm were visible to the naked eye under a microscope. Cell colonies were observed under a microscope (DMi1, Leica, Wetzlar, Germany), counted and photographed.

### 2.3 Transwell migration and invasion assays

For the invasion assay, the upper chamber of the Transwell chamber (8 μm in diameter, CLS3422, Sigma-Aldrich, St. Louis, MO, USA) was pre-coated with 50 μL of Matrigel (diluted 1: 8 with serum-free RPMI 1640 medium) and incubated for 1 h to solidify. BL cells were harvested, washed twice with PBS, and resuspended in serum-free RPMI 1640 medium at a density of 6×10^4^ cells/mL. Then, 100 μL of the cell suspension was added to the upper chamber, and 700 μL of serum-containing medium was added to the lower chamber. After 48 h of culture, the non-invading cells on the upper surface of the membrane were gently removed with a cotton swab. The invading cells were fixed with 4% paraformaldehyde for 20 min, stained with 0.1% crystal violet (C0121, Beyotime, Shanghai, China) for 30 min, and washed with PBS. Images were captured from five randomly selected fields under a light microscope (DMi1, Leica, Wetzlar, Germany), and the number of invading cells was counted.

For the migration assay, the same procedure was followed except that the Transwell chamber was not coated with Matrigel. BL cells were seeded in the upper chamber under serum-free conditions, and the lower chamber contained 700 μL of serum-containing medium. After 48 h of incubation, the migrated cells on the lower membrane surface were fixed, stained, and counted as described above.

### 2.4 Flow cytometry experiment

BL cells were digested with trypsin without EDTA to collect cells, added 5 μL PI and 5 μL Annexin V-FITC (E-CK-A211, Elabscience, Wuhan, China) dye solution, gently blown, incubated for 15 min. Flow cytometry was used to detect double positive cell proportion.

After transfection, BL cells were digested with trypsin, and the cells were collected. The cells were resuspended in precooled 70% ethanol and fixed overnight at 4°C. The next day, cells were incubated with PI staining solution containing RNase A (C1731, Beyotime) for 30 min in dark to digest RNA and ensure that PI only binds to DNA. Flow cytometry analyzed cell cycle, FlowJo 10.8.1 software was used to analyze the proportion of cells in each period (25).

### 2.5 Chromatin immunoprecipitation-qPCR

ChIP is a technique used to study the interaction between protein and DNA. The basic principle is to use formaldehyde to reversibly cross-link intracellular DNA with binding proteins. After ultrasonic fragmentation, the chromatin is fragmented, and specific antibodies are used to enrich the DNA fragments that bind to the target protein. Through de-crosslinking and DNA purification, the enrichment of specific gene promoter regions is finally detected by qPCR. This method can quantitatively evaluate the binding sites and binding strength of transcription factors on the genome. The ChIP experiment was performed using the SimpleChIP^®^ Plus Sonication Chromatin IP Kit (56383, Cell Signaling Technology, CST, Danvers, MA, USA). BL cells were fixed with 1% formaldehyde for 10 min and treated with 50 mM glycine for 10 min. After washing with PBS twice, the cells were lysed on ice for 10 min with cell lysis buffer containing protease inhibitor, and the nuclear precipitation was collected by centrifugation. The nucleus was resuspended in the nuclear lysis buffer, and the chromatin was fragmented to about 100–500 bp by ultrasonication. After centrifugation (16000 r/min, 10 min, 4°C), the chromatin was incubated with anti-MYC antibody (ChIP grade, 9402, CST) for immunoprecipitation to enrich the DNA fragments bound to MYC protein, and the immune complex was recovered by Protein A magnetic beads (73778, CST). At the same time, 2% of the chromatin sample was retained as the Input control. The magnetic bead complexes were washed once with low-salt washing buffer, high-salt washing buffer, LiCl washing buffer and TE buffer, 5 min each time. After washing, the elution buffer was added to elute at room temperature for 15 min, repeated once and the eluent was combined. 5 M NaCl (final concentration of 200 mM) and protease K were added to the eluent and incubated at 65°C for 2 h to decrosslink. Subsequently, the purified DNA was recovered by DNA purification column. Finally, the purified DNA was used as a template. Specific primers designed for the promoter regions of target genes (including carbamoyl-phosphate synthetase 2, aspartate transcarbamylase, dihydroorotase (CAD), cyclin-dependent kinase (CDK) 4, lactate dehydrogenase A (LDHA), nucleolin (NCL), pyruvate kinase M2 (PKM2) and Hairy and enhancer of split 1 (HES1)) were used for qPCR. Reaction procedure: 95°C for 10 min; after 40 cycles of denaturation at 95°C for 15 s and annealing/extension at 60°C for 1 min, the enrichment factor of each gene promoter region was calculated by 2^-ΔΔCt^ method to evaluate the effect of THBS2 deletion on the binding ability of MYC to these target gene promoters. All ChIP experiments used IgG-matched species as a control (26). The qPCR primer sequences was shown in **Supplementary Table 1**.

### 2.6 Co-immunoprecipitation

CO-IP is a classical method for studying protein-protein interactions. The basic principle is to use specific antibodies to bind to the target protein, capture the antibody-protein complex by Protein A/G magnetic beads, co-precipitate the target protein and its interacting proteins from the cell lysate, and then detect the co-precipitated protein by Western blot. This method can be used to verify whether there is an endogenous binding between the two proteins. BL cells were lysed in CO-IP buffer (containing protease inhibitor (5817, CST), 1 mM trichostatin A (HY-15144, MedChemExpress) and 5 mM nicotinamide (HYB0150, MedChemExpress)) at 4°C for 30 min to retain the natural conformation and interaction of the protein. The lysate was centrifuged at 12000 r/min for 15 min at 4°C, and the supernatant was collected. The total protein concentration of the lysate supernatant was determined by BCA method. The same amount of total protein was added to anti-MYC antibody (ChIP grade, 9402, CST), anti-MYC associated factor X (MAX) antibody (ChIP grade, 17471, CST) and control IgG, respectively, and incubated overnight at 4°C. Protein A/G magnetic beads were added and incubated to capture the antibody-protein complex. The magnetic beads complex was collected and washed three times with low-salt washing buffer and high-salt washing buffer, respectively, for 5 min each time. Finally, wash with PBS once. After washing, 30 μL 1 × SDS loading buffer was added, and the protein complex was eluted by boiling at 95°C for 10 min. The magnetic beads were separated by magnetic frame, and the supernatant was collected for Western blot detection. At the same time, 10% of the original lysate was retained as Input control. Finally, MAX and MYC proteins were detected by Western blot (26).

### 2.7 Tumor stem cell sphere formation experiment

After the treatment of BL cells, the single cell suspension was prepared and inoculated into a 6-well plate with ultra-low adsorption (3471, Corning, Corning, NY, USA). The cells were suspended in DMEM/F12 medium. Add 20 ng/mL human recombinant epidermal growth factor (hrEGF), 20 ng/mL human recombinant basic fibroblast growth factor (hrbFGF), 1% non-essential amino acids, 1% GlutaMAX, 2% B27 supplement (17504044, Invitrogen) and 1% N-2 supplement (17502048, Invitrogen). Subsequently, the cells were cultured in a cell incubator for 7–14 days, and fresh medium was supplemented every 2–3 days. At the end of culture, the number of cell spheres was observed and counted under a microscope, and the size and morphology of the cell spheres were photographed to evaluate the stem cell self-renewal ability of BL cells (26).

### 2.8 *In vivo* experiments

SPF male severe combined immunodeficiency (SCID) mice (4 weeks old, 15–18 g) were purchased from Beijing Vital River Laboratory Animal Technology Co., Ltd. After 1 week of adaptive feeding in a standard SPF barrier environment, the mice were used for experimental research. The temperature of the feeding environment was 22 ± 2°C, the humidity was 55%-65%, and the light and dark alternated every 12 h. During the period, free feeding and drinking water. This study has been approved by the Ethics Committee of First Hospital of Quanzhou Affiliated to Fujian Medical University (Approval No.: FJ--2025-04).

RAJI cells treated with stable knockdown of THBS2 and its control were collected and resuspended with medium (1×10^8^ cells/mL). 50 μL cell suspension was subcutaneously injected into the left armpit of SCID mice, and the pinhole was gently pressed after injection to avoid cell leakage. The tumor growth was monitored regularly. After the tumor volume grew to about 100 mm^3^, the mice were randomly divided into 5 groups (n=6): Control group, si-NC group, si-THBS2 group, si-THBS2 + PI3K activator 740Y-P group and si-THBS2 + oe-MYC group. Among them, the si-THBS2 + oe-MYC group was subcutaneously injected with RAJI cells stably co-transfected with si-THBS2 and oe-MYC plasmids. The mice in the si-THBS2 + 740Y-P group were intraperitoneally injected with 10 mg/kg 740Y-P every day for 4 weeks (27, 28). During this period, the long diameter (a) and short diameter (b) of the tumor were measured, and the tumor volume was calculated according to the formula V = 1/2×a×b^2^, and the dynamic changes were recorded. After 4 weeks of treatment, the mice were placed in a CO_2_ euthanasia device, and CO_2_ was introduced at a flow rate of 25% of the volume in the box per minute for 5 minutes until the mouse’s breathing and heartbeat stopped to confirm death. The tumor tissue was completely stripped under sterile conditions. After removing the surrounding adhesion tissue, the tumor weight was weighed with an electronic balance, and the data were recorded and photographed.

### 2.9 Western blot

BL cells and tumor tissues were collected, RIPA lysate was added, and placed for 30 min. After centrifugation, the supernatant was collected as the total protein solution. The BCA kit was used to quantify the protein, and the appropriate volume was taken. The loading buffer was added at a ratio of 1: 5, and the sample was denatured in a boiling water bath at 100°C for 10 min. The loading system was configured, 30 μg protein was loaded, and the target protein was separated by SDS-PAGE electrophoresis. The PVDF membrane was used for membrane transfer, blocked with 5% skim milk for 2 h, and then incubated with primary antibody dilution at 4°C overnight, and then incubated with the corresponding secondary antibody dilution for 2 h. ECL kit (HY-K1005, MedChemExpress) was used for chemiluminescence detection, and gel imaging system (Geldoc Go, Bio-Rad, Shanghai, China) was used to detect protein bands. The ratio of the target protein to the GAPDH was used as the relative expressions. All Western blot experiments were independently repeated three times with similar results, and representative images were shown.

The primary antibodies were purchased from Abcam (Cambridge, UK): THBS2 (ab89805, 1: 1000), Epithelial cadherin (E-cadherin, ab40772, 1: 50000), Neural cadherin (N-cadherin, ab18203, 1: 1000), vimentin (ab8069, 1: 1000), Snail (ab216347, 1: 1000), B-cell lymphoma 2 (Bcl2, ab117115, 1: 2000), Bcl-2 associated X protein (Bax, ab243140, 1: 500), cleaved-caspase3 (c-caspase3, ab32042, 1: 500), caspase3 (ab32351, 1: 5000), CDK2 (ab32147, 1: 10000), CDK4 (ab137675, 1: 3000), CDK6 (ab151247, 1: 3000), Cyclin D1 (ab239794, 1: 1000), PI3K (ab191606, 1: 1000), p-PI3K (ab278545, 1: 1000), AKT (ab8805, 1: 2000), p-AKT (ab38449, 1: 1000), c-MYC (ab32072, 1: 1000), SRY-box transcription factor 2 (SOX2, ab93689, 1: 100), octamer-binding transcription factor 4 (OCT4, ab181557, 1: 1000), and GAPDH (ab181602, 1: 10000).

### 2.10 Immunohistochemistry

The tumor tissue samples were cut into 4 μm thick sections. After the paraffin section of the tumor tissue was baked, xylene was dewaxed and gradient ethanol was hydrated. After incubation with 3% H_2_O_2_ for 25 min in the dark, endogenous peroxidase was inhibited. After washing with PBS, the sections were placed in 5% bovine serum albumin (BSA, V900933, Sigma-Aldrich) for 30 min. Ki67 (ab279653, 1: 50, Abcam) and proliferating cell nuclear antigen (PCNA, ab18197, 1: 500, Abcam) primary antibodies were added and incubated overnight at 4°C. The next day, the first antibody was discarded, and the second antibody (ab6721,1: 1000, Abcam) was added dropwise, incubated for 2 h, PBS rinsed, DAB (DA1010, Solarbio) coloration, hematoxylin nuclear re-staining. Under the microscope, the positive signal was brownish yellow granules, and the expression of the protein was analyzed.

### 2.11 TUNEL staining

Paraffin sections of tumor tissue were de-waxed to water, DNase-free protease K (20 μg/mL, ST532, Beyotime) was used to repair antigen, and membrane rupture fluid was used to penetrate the membrane. After treatment, TUNEL reaction solution (C1086, Beyotime) was added and incubated for 90 min. After washing, the nuclei were re-stained with DAPI (C0060, Solarbio) for 5 min. The staining was observed under a fluorescence microscope, and the proportion of positive cells was counted by Image J software to statistically analyze apoptosis.

### 2.12 Statistical analysis

Data were presented as mean ± SD and analyzed with SPSS 27.0 software. The Shapiro-Wilk and Levene tests were used to verify normal distribution and variance homogeneity. The Student’s t-test was used for comparison between two groups, and One-way ANOVA analysis and Tukey *post-hoc* test were used for comparisons among multiple groups. A *p* value < 0.05 indicated statistically significant differences. Graphpad Prism 9.0 software was used for chart drawing.

## 3 Results

### 3.1 THBS2 was up-regulated in BL cells and promoted growth, migration and EMT

**Figure 1:**
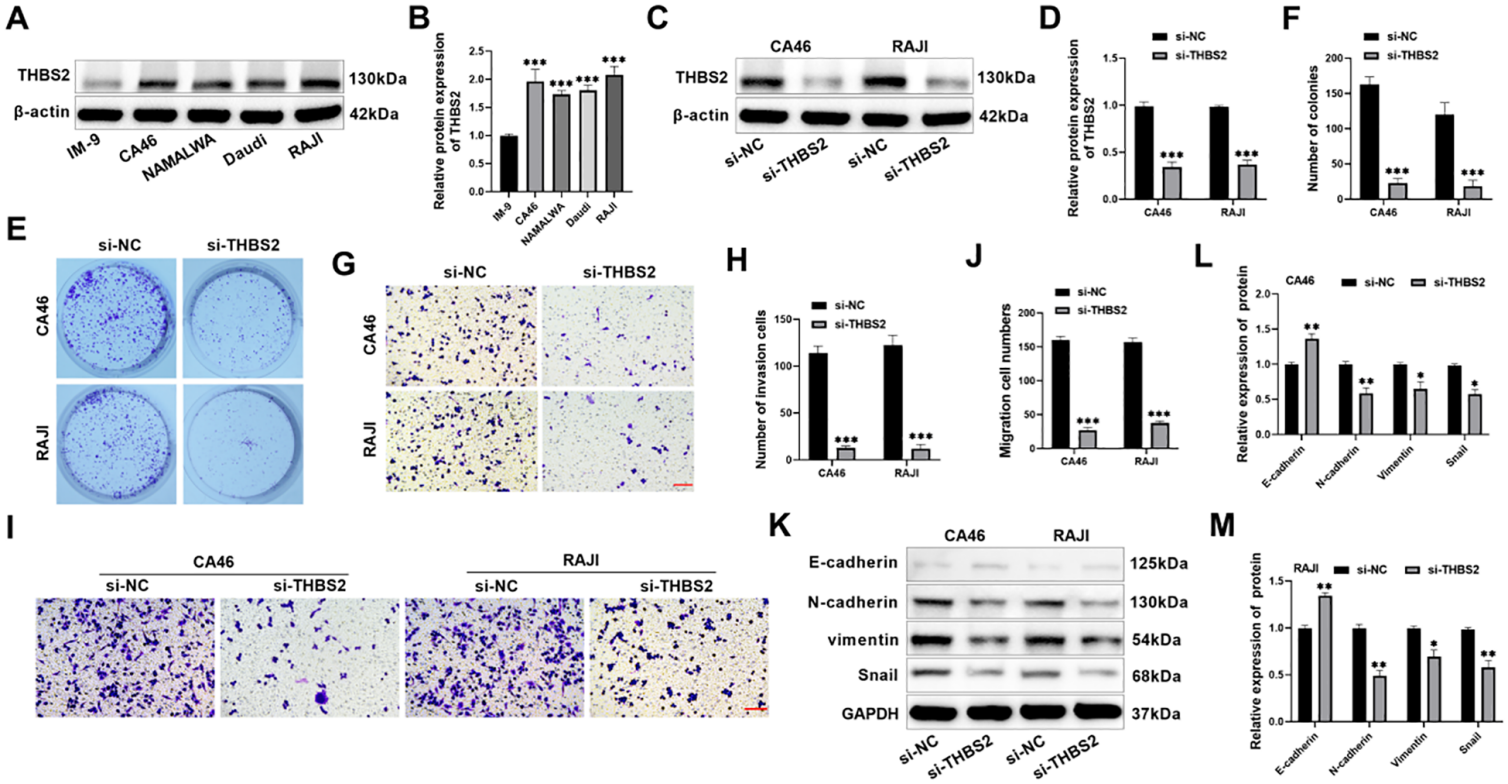
THBS2 was up-regulated in BL cells and promoted cancer cell proliferation, migration and EMT. **(A, B)** WB was used to detect THBS2 protein in normal human B lymphocytes (IM-9) and four BL cells. THBS2 protein was highly expressed in BL cells. **(C, D)** WB verified the knockdown efficiency of THBS2. **(E, F)** Representational figures and analysis of colony formation experiments. THBS2 knockdown significantly reduced the number of colonies. **(G-J)** Transwell assays detected migration and invasion ability. They were significantly reduced after THBS2 knockdown (×20, 100 μm). **(K-M)** WB detected EMT-related markers in CA46 and RAJI cells. Knockdown of THBS2 significantly elevated E-cadherin protein and declined N-cadherin, vimentin and Snail expressions. n=3, *p < 0.05, **p < 0.01, ***p < 0.001 *vs* IM-9/si-NC group.

THBS2 is a prognostic gene for BL. In order to investigate THBS2 expression characteristics and functions in BL, this study first detected THBS2 expressions in different BL cells. THBS2 protein was highly expressed in BL cells compared to human normal B lymphocyte IM-9. Among them, THBS2 protein was highly expressed in CA46 and RAJI cells (**Figures 1A, B**), suggesting that THBS2 might be important for the development of BL. To further verify its function, we transfected si-THBS2 into CA46 and RAJI cells with high expression of endogenous THBS2. WB verification results showed that si-THBS2 transfection significantly reduced THBS2 protein level, indicating that the knockdown efficiency was reliable (**Figures 1C, D**). The colony number was significantly declined with si-THBS2 transfection, with a significant decline in both the number and size of colonies (**Figures 1E, F**), indicating that knockdown of THBS2 effectively inhibited the growth of BL cells. The number of migration and invasion cells decreased markedly with THBS2 knockdown (**Figures 1G–J**), suggesting that THBS2 knockdown weakened the migration and invasion abilities. We examined EMT-related marker levels. After knockdown of THBS2 in CA46 and RAJI cells, E-cadherin expression was significantly up-regulated, N-cadherin, vimentin and transcription factor Snail expressions were significantly down-regulated (**Figures 1K–M**), indicating that THBS2 knockdown reversed the EMT process. In summary, THBS2 expressions were positively correlated with cell proliferation, invasion and migration, and were involved in regulating the malignant progression of BL by promoting EMT process.

### 3.2 Down-regulation of THBS2 promoted apoptosis and arrested cell cycle

**Figure 2:**
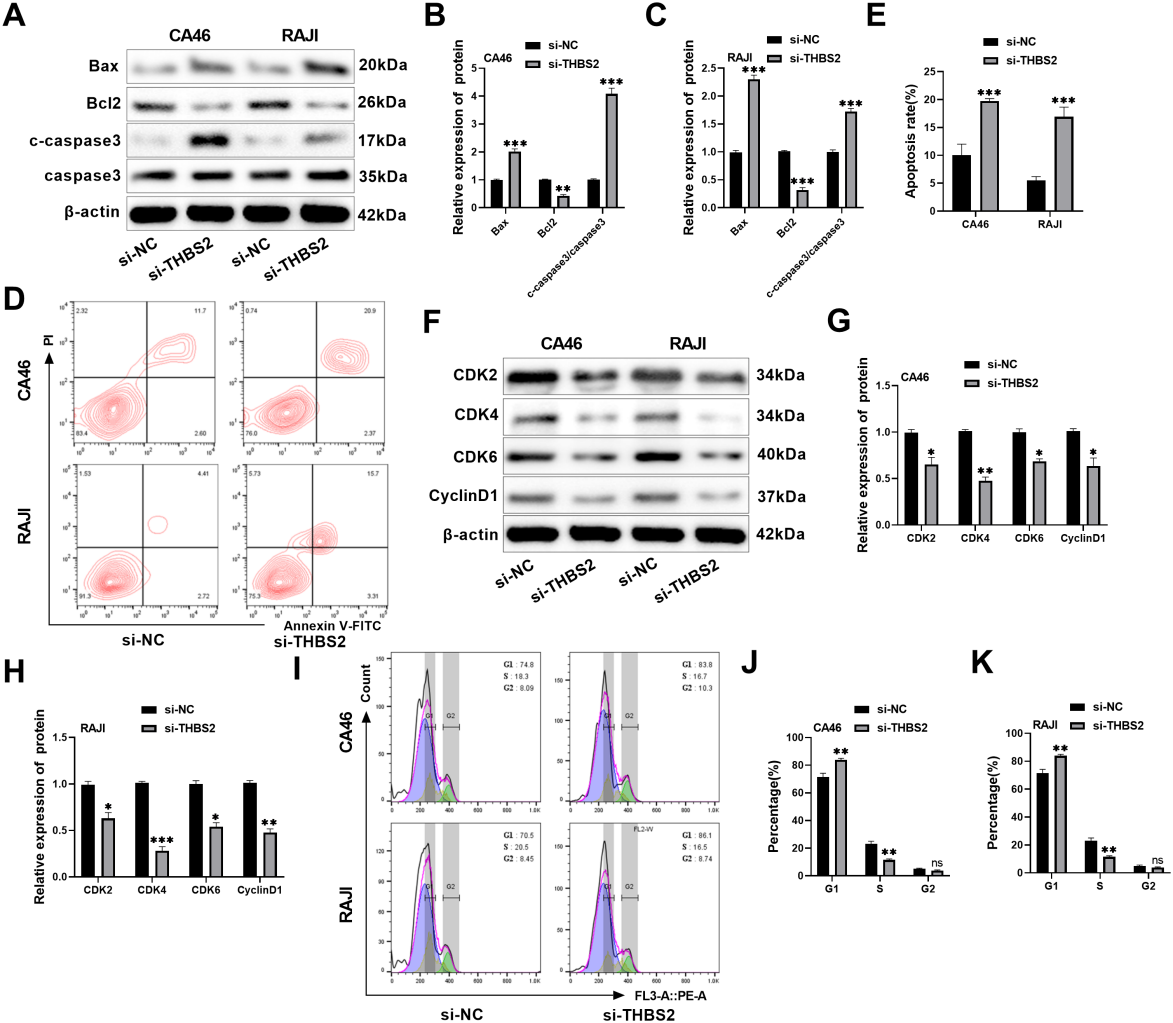
Down-regulation of THBS2 promoted apoptosis of BL cells and arrested the cell cycle in G0/G1 phase. **(A-C)** WB was used to detect apoptotic proteins in CA46 and RAJI cells. Knockdown of THBS2 significantly elevated Bax and Cleaved-caspase3 proteins, and significantly declined Bcl2 protein. **(D, E)** Flow cytometry detected CA46 and RAJI cell apoptosis. Apoptosis rates were significantly increased upon THBS2 knockdown. **(F-H)** WB detected cell cycle proteins (CDK2, CDK4, CDK6 and Cyclin D1). They were significantly reduced after THBS2 knockdown. **(I-K)** Flow cytometry detected cell cycle. THBS2 knockdown induced G0/G1 phase arrest. n=3, *p < 0.05, **p < 0.01, ***p < 0.001 *vs* si-NC group.

To explore the potential molecular mechanism of THBS2 in regulating BL cell proliferation, this study further examined the effect of THBS2 knockdown. First, the apoptotic proteins were detected by WB. After knockdown of THBS2, the protein expressions of Bax and c-caspase3 were significantly elevated, while the protein expression of Bcl2 was significantly decreased (**Figures 2A–C**), indicating that THBS2 knockdown activated mitochondrial-mediated apoptosis pathway. Subsequently, the apoptosis rates of CA46 and RAJI cells were significantly raised (**Figures 2D, E**), which further confirmed that knockdown of THBS2 could effectively induce apoptosis of BL cells. Abnormal cell cycle regulation is an important feature of infinite proliferation of tumor cells. After knockdown of THBS2 in CA46 and RAJI cells, CDK2, CDK4, CDK6 and Cyclin D1 expressions were markedly diminished (**Figures 2F–H**), suggesting that THBS2 deficiency might lead to cell cycle arrest in G0/G1 phase. To further verify this finding, after knockdown of THBS2, cells in G0/G1 phase increased significantly, while cells in S phase decreased correspondingly (**Figures 2I–K**). In conclusion, THBS2 knockdown effectively promoted the apoptosis of BL cells and induced cell cycle arrest.

### 3.3 Down-regulation of THBS2 inhibited the PI3K/AKT/c-MYC axis

**Figure 3:**
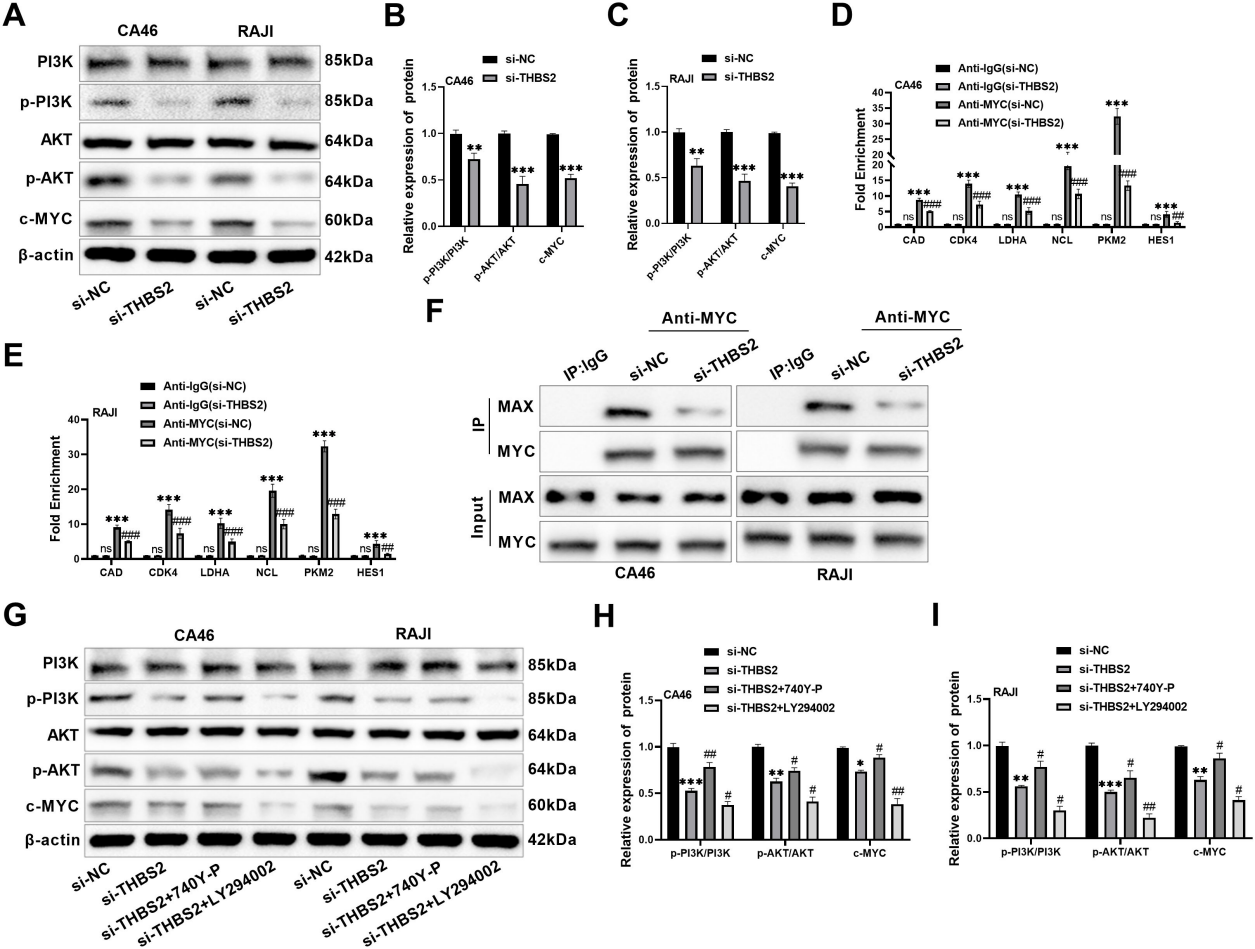
THBS2 down-regulation inhibited the PI3K/AKT/c-MYC axis. **(A-C)** WB detected the PI3K/AKT pathway and c-MYC protein within CA46 and RAJI cells. Knockdown of THBS2 significantly down-regulated p-PI3K, p-AKT and c-MYC proteins. **(D, E)** ChIP-qPCR detected the enrichment levels of MYC in its target gene promoters in CA46 and RAJI cells. They were significantly reduced after knockdown of THBS2. **(F)** CO-IP assays detected the interaction between MYC and MAX. The deletion of THBS2 weakened the dimerization of MYC/MAX. **(G-I)** On the basis of THBS2 knockdown, CA46 and RAJI cells were treated with 740Y-P and LY294002, respectively. WB was used to detect the PI3K/AKT/c-MYC axis. n=3, *p < 0.05, **p < 0.01, ***p < 0.001 *vs* si-NC/Anti-lgG(si-NC) group; #*p* < 0.05, ##*p* < 0.01, ###*p* < 0.001 *vs* Anti-MYC(si-NC)/si-THBS2 group.

The proteins of the PI3K/AKT/c-MYC pathway are closely related to the malignant progression of BL. This study evaluated whether THBS2 knockdown affected the PI3K/AKT signaling pathway and c-MYC expression. After knockdown of THBS2, p-PI3K and p-AKT expressions were significantly decreased, and c-MYC protein level was also significantly down-regulated (**Figures 3A–C**), indicating that THBS2 deficiency suppressed the phosphorylation of the PI3K/AKT pathway and the protein expression of c-MYC. The cancer-promoting function of c-MYC depends on the formation of heterodimers with MAX, which in turn activates transcription via binding to target gene promoters. In order to explore whether THBS2 knockdown affects the transcriptional activity of c-MYC, ChIP-qPCR assessed the binding capacity of c-MYC to downstream gene promoters. After knockdown of THBS2, the enrichment levels of c-MYC in the promoter regions of CAD, CDK4, LDHA, NCL, PKM2 and HES1 were significantly reduced (**Figures 3D, E**), indicating that THBS2 deficiency weakened c-MYC binding to these target gene promoters. The results showed that after THBS2 knockdown, the amount of MAX protein detected from the MYC immunoprecipitation complex was significantly reduced (**Figure 3F**), confirming that THBS2 deletion weakened the formation of MYC/MAX dimers, which might be the direct reason for the decrease of c-MYC transcriptional activity. In order to elucidate its role in regulating c-MYC expression by THBS2, we used 740Y-P and LY294002 to treat CA46 and RAJI cells on the basis of THBS2 knockdown. Compared with knockdown of THBS2, the addition of 740Y-P partially restored p-PI3K, p-AKT and c-MYC protein expressions, the addition of LY294002 further inhibited these protein expressions (**Figures 3G–I**). This indicated that THBS2 knockdown down-regulated c-MYC expression by suppressing the PI3K/AKT pathway, and this pathway played a key role in this regulatory process. In summary, THBS2 knockdown declined c-MYC protein via suppressing the PI3K/AKT axis, while weakening the formation of MYC/MAX dimers and the binding ability of c-MYC to bind to target gene promoters, thereby inhibiting c-MYC transcriptional activity.

### 3.4 Down-regulation of MYC suppressed BL cell stem cell-like properties

**Figure 4:**
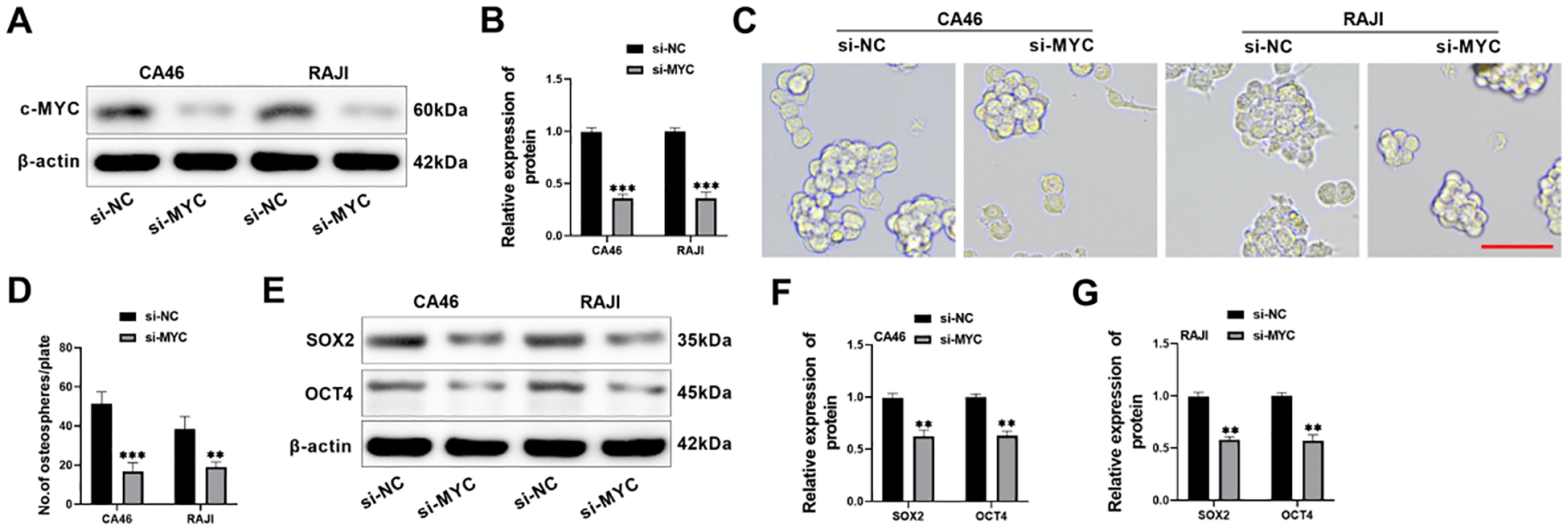
Down-regulation of MYC inhibited BL cell stem cell-like properties. **(A, B)** WB detected c-MYC protein. si-MYC markedly declined c-MYC protein. **(C, D)** Representative images and analysis of tumor stem cell sphere formation experiments in CA46 and RAJI cells. Knockdown of MYC significantly reduced the number of stem cell spheres formed by cells (×40, 50 μm). **(E-G)** WB was used to detect stem cell markers (SOX2 and OCT4) in CA46 and RAJI cells. Their expression levels were significantly reduced after MYC knockdown. n=3, **p < 0.01, ***p < 0.001 *vs* si-NC group.

Tumor stem cells play a critical role in tumor growth, treatment resistance, and recurrence. The above results have confirmed that THBS2 knockdown can down-regulate c-MYC expression, and c-MYC, as one of the core transcription factors (OCT4, SOX2, KLF4, and MYC), is vital for maintaining the characteristics of tumor stem cells (29). To explore whether THBS2 regulates the stemness of BL cells through c-MYC, this study further investigated whether MYC knockdown affected the stem-like characteristics. First, after transfection of si-MYC, c-MYC protein was significantly decreased (**Figures 4A, B**), indicating that the knockdown efficiency was reliable. Subsequently, the effect of MYC knockdown on the self-renewal abilities was evaluated. Knockdown of MYC significantly reduced stem cell spheres formed by cells, and significantly reduced the number and diameter of stem cell spheres (**Figures 4C, D**), indicating that knockdown of MYC effectively inhibited the stem cell sphere formation ability of BL cells and weakened their self-renewal characteristics. The characteristics of cancer stem cells were synergistically regulated by a series of stem transcription factors. After knockdown of MYC in CA46 and RAJI cells, SOX2 and OCT4 expressions were significantly down-regulated (**Figures 4E–G**). In summary, MYC knockdown significantly inhibited BL cell stem cell-like characteristics.

### 3.5 THBS2 promoted BL cell malignant progression through c-MYC

**Figure 5:**
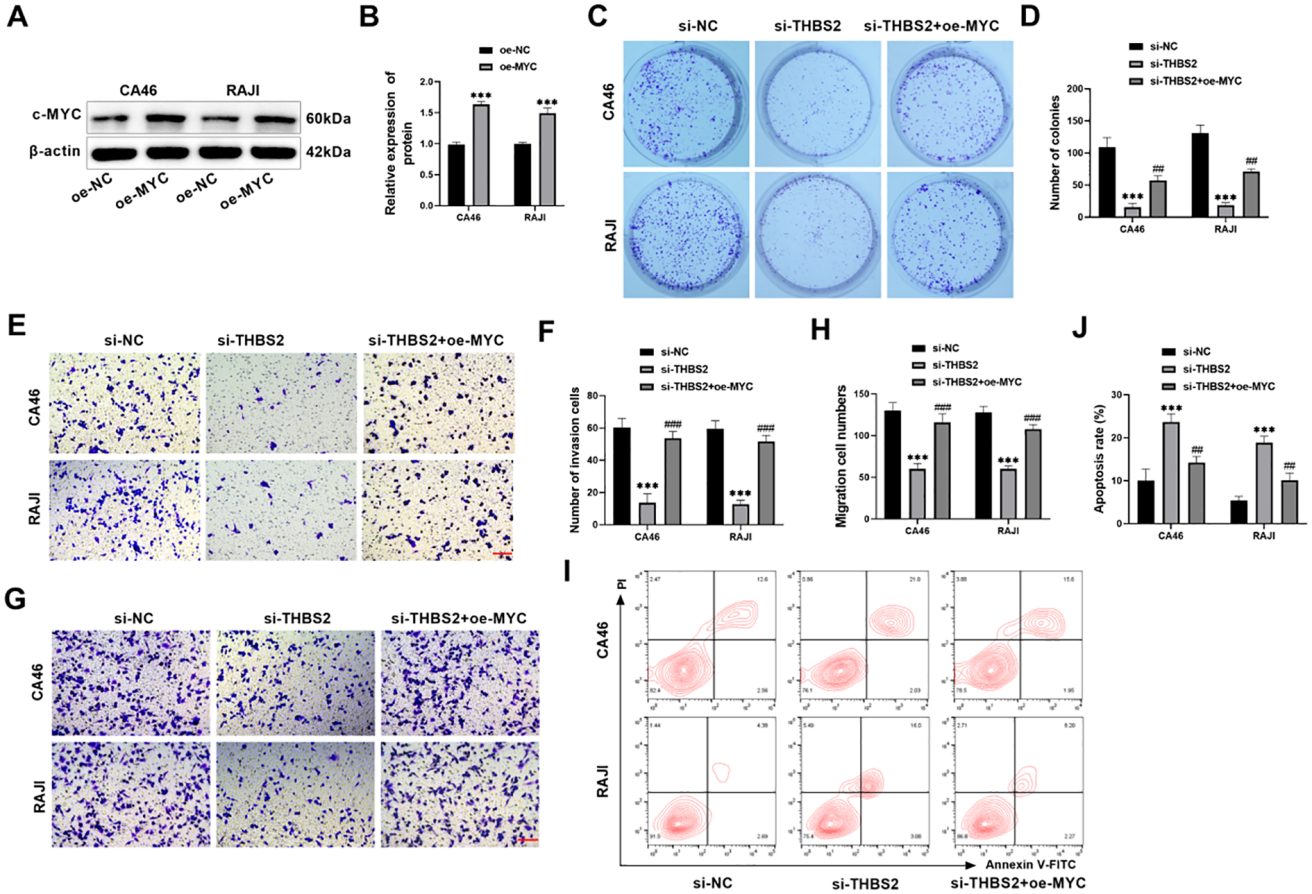
THBS2 promoted BL cell malignant progression through c-MYC. **(A, B)** WB detected c-MYC level within CA46 and RAJI cells. oe-MYC significantly increased the expression level of c-MYC protein. **(C, D)** Representative images and analysis of colony formation experiments. Overexpression of MYC significantly increased the number of colonies. **(E-H)** Transwell assays detected the migration and invasion abilities. It was markedly elevated with MYC overexpression (×20, 100 μm). **(I, J)** Flow cytometry detected the apoptosis of CA46 and RAJI cells. It was significantly reduced after MYC overexpression. n=3, *p < 0.05, ***p < 0.001 *vs* oe-NC/si-NC group; ##*p* < 0.01, ###*p* < 0.001 *vs* si-THBS2 group.

In order to further clarify whether THBS2 regulates BL cell malignant progression through c-MYC, functional rescue experiments were carried out in this study. Firstly, the efficiency of MYC overexpression was verified by WB. c-MYC protein levels were markedly increased in the oe-MYC group (**Figures 5A, B**), indicating that the overexpression efficiency was reliable and could be used for subsequent rescue experiments. Subsequently, compared with THBS2 knockdown, the colony formation ability of si-THBS2 + oe-MYC group was significantly restored (**Figures 5C, D**), the number of migration and invasion cells was significantly increased (**Figures 5E–H**), but the apoptosis rate was significantly decreased (**Figures 5I, J**), indicating that overexpression of c-MYC partially reversed the tumor malignant phenotype suppression induced by THBS2 knockdown. Moreover, compared with the si-NC group, the malignant phenotype of the cells in the si-NC + oe-MYC group was significantly promoted, and the apoptosis rate was significantly reduced. These results confirmed that THBS2 promoted the malignant progression of BL cells by up-regulating c-MYC expression, and c-MYC was a key effector molecule downstream of THBS2.

### 3.6 Downregulation of THBS2 inhibited tumor growth *in vivo*

**Figure 6:**
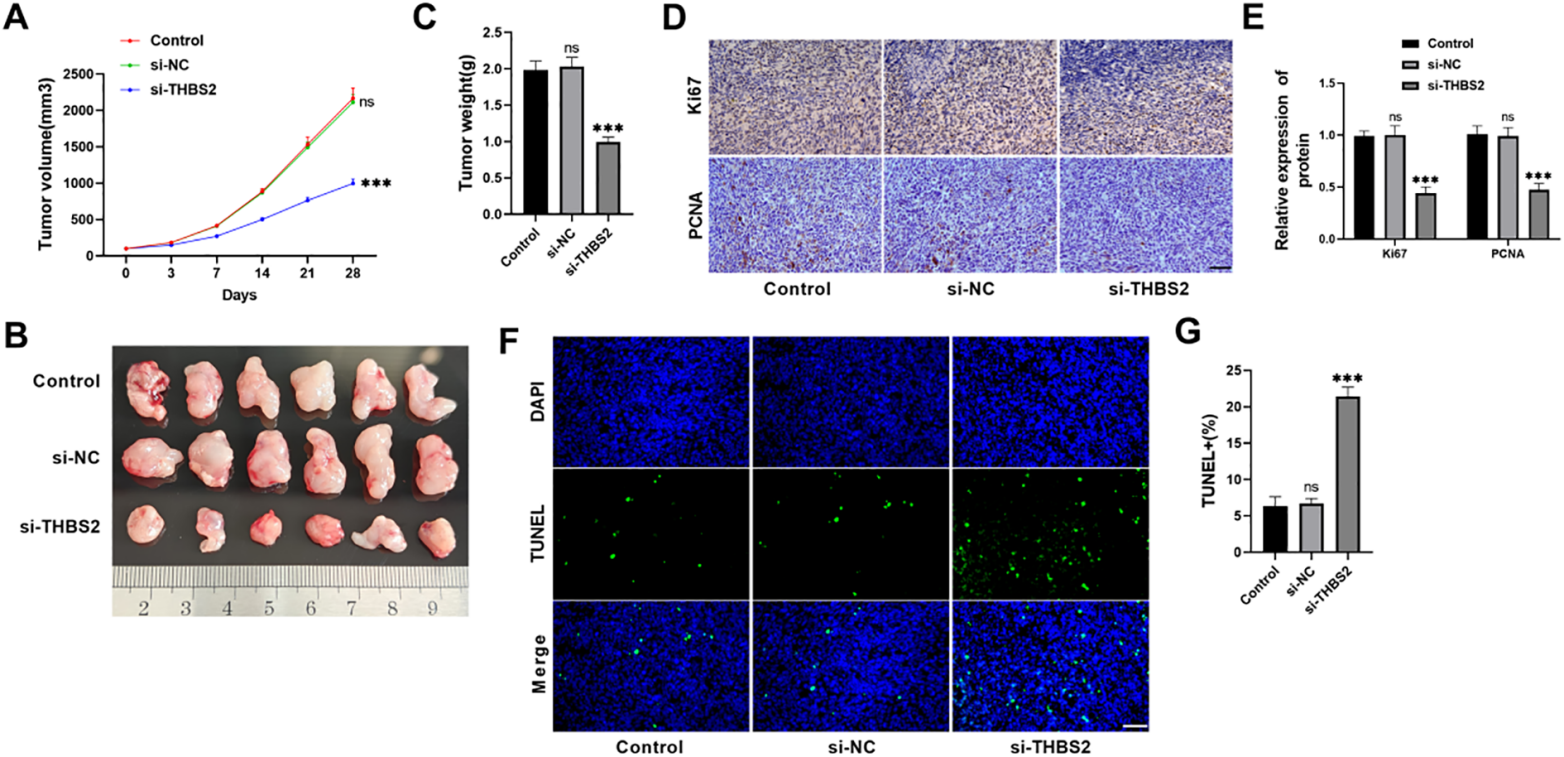
Downregulation of THBS2 suppressed tumor growth. **(A)** Tumor volume growth curve of SCID mice subcutaneous xenografts inoculated with Control, si-NC or si-THBS2 RAJI cells. THBS2 knockdown significantly reduced tumor volume. **(B, C)** Representative images and tumor weight of subcutaneous transplanted tumors in each group on the 28th day. THBS2 knockdown significantly reduced tumor weight. **(D, E)** Immunohistochemistry detected Ki67 and PCNA expressions. It was significantly reduced after THBS2 knockdown (×40, 50 μm). **(F, G)** TUNEL staining assessed and analyzed apoptotic cells. Knockdown of THBS2 significantly increased the TUNEL positive rate (×40, 50 μm). n=6, ns, *p* > 0.05 *vs* Control group; ***p < 0.001 *vs* si-NC group.

THBS2 knockdown suppressed BL cell growth, induced apoptosis and weakened stem cell-like properties. To further verify the cancer-promoting effects of THBS2, subcutaneous xenograft models of RAJI cells were constructed in this study. RAJI cells stably transfected with si-THBS2 and si-NC were inoculated into SCID mice, and tumor growth was monitored regularly. The results of dynamic monitoring of tumor volume showed that the tumor volume of si-THBS2 group began to decrease from the 7th day after inoculation, and the difference gradually increased with time. The tumor volumes of the si-THBS2 group were significantly smaller (**Figure 6A**); the gross image of the tumor stripped showed that the tumor size was significantly reduced (**Figure 6B**), indicating that THBS2 knockdown effectively inhibited tumor growth *in vivo*. Moreover, knockdown of THBS2 significantly reduced the average tumor weight (**Figure 6C**), consistent with the volume change trend. The results of immunohistochemistry showed that Ki67 and PCNA positive cell proportion was markedly decreased after THBS2 knockdown (**Figures 6D, E**), indicating that THBS2 knockdown could inhibit tumor proliferation. In addition, TUNEL-positive cell proportion was markedly increased after THBS2 knockdown (**Figures 6F, G**), indicating that THBS2 knockdown induced apoptosis in tumor cells. In summary, THBS2 knockdown significantly inhibited the growth of subcutaneous xenografts of BL cells.

### 3.7 Down-regulation of THBS2 interfered with the cell cycle via the PI3K/AKT/c-MYC axis

**Figure 7:**
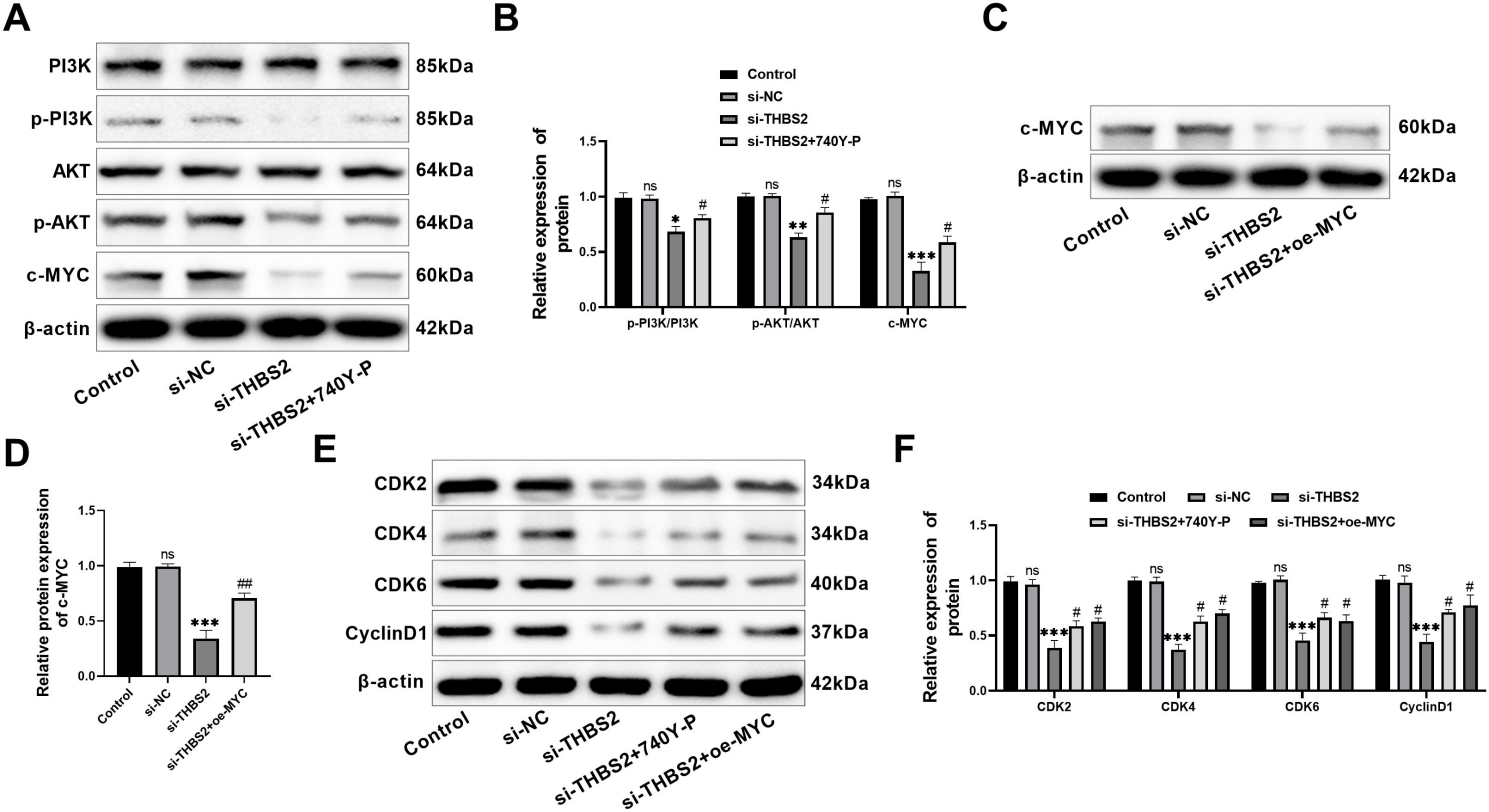
Down-regulation of THBS2 interfered with the cell cycle *in vivo* through the PI3K/AKT/c-MYC axis. **(A, B)** WB detected the PI3K/AKT/c-MYC axis. Knockdown of THBS2 significantly down-regulated p-PI3K, p-AKT and c-MYC proteins. **(C, D)** WB detected the transfection efficiency of c-MYC after transfection of si-THBS2 or oe-MYC. **(E, F)** WB detected cell cycle proteins in tumor tissues. They were significantly reduced after knockdown of THBS2. n=6, ns, *p* > 0.05 *vs* Control group; *p < 0.05, **p < 0.01, ***p < 0.001 *vs* si-NC group; #*p* < 0.05, ##*p* < 0.01 *vs* si-THBS2 group.

Firstly, the PI3K/AKT pathway activation state was verified. After knockdown of THBS2, p-PI3K and p-AKT expressions in tumor tissues were significantly reduced, and c-MYC protein was also markedly declined. Combined treatment with 740Y-P markedly restored these protein levels (**Figures 7A, B**), confirming that 740Y-P effectively activated the PI3K/AKT/c-MYC axis *in vivo*. Moreover, compared with knockdown of THBS2, oe-MYC significantly increased c-MYC protein expression (**Figures 7C, D**), indicating that oe-MYC effectively overexpressed c-MYC *in vivo* and could be used for subsequent mechanism verification. Subsequently, WB results showed that knockdown of THBS2 significantly reduced CDK2, CDK4, CDK6 and Cyclin D1 expressions in tumor tissues; compared with THBS2 knockdown, the expression of the above cyclins was significantly restored after combined use of 740Y-P or oe-MYC (**Figures 7E, F**), indicating that this pathway activation or c-MYC overexpression could partially reverse the cycle arrest caused by THBS2 knockdown. In summary, THBS2 knockdown induced cell cycle arrest, and the mechanism involved suppression of the PI3K/AKT pathway activity and down-regulation of c-MYC expression.

## 4 Discussion

This study systematically revealed the tumor-promoting effect of THBS2 in BL and its molecular mechanism. THBS2 is overexpressed in BL cells and promotes tumor malignant progression, EMT and stem cell-like properties by activating the PI3K/AKT/c-MYC axis, while inhibiting apoptosis and interfering with cell cycle progression.

The function of THBS2 within solid tumors is well-established, but its function in lymphoid hematopoietic system tumors has rarely been reported. Our results are highly consistent with the findings in solid tumors. THBS2 is highly expressed in a variety of BL cells, suggesting that THBS2 may be vital for BL development. Our findings are highly consistent with recent bioinformatics studies. Through the analysis of transcriptome sequencing data of BL patients in Africa, Doughan et al. (23) found that THBS2 is one of the six hub genes significantly related to the overall survival of BL patients. Furthermore, the differential genes in BL are involved in biological processes such as PI3K/AKT signaling pathway, cell migration, and external stimulus response through pathway enrichment analysis.

Invasion and migration are vital processes of tumor metastasis. The stronger the invasion and migration, the more likely the tumor cells are to metastasize. Tumor cells can escape apoptosis through malignant biological behaviors such as abnormal proliferation, invasion or metastasis (30). Therefore, induction of tumor cell apoptosis is the most effective means of clinical treatment of tumor diseases. Bcl2 family proteins are vital for regulating the process of apoptosis (31). In this study, knockdown of THBS2 inhibited the growth and migration of BL cells, and elevated pro-apoptotic proteins, thereby promoting apoptosis. The migration of cancer cells is also regulated by EMT. In this process, cell adhesion factor E-cadherin protein is decreased, the keratin cytoskeleton is changed into a wavy cytoskeleton, and N-cadherin protein is increased, which promotes cell penetration of intercellular connections and enhances cell invasion (32, 33). This study found that after THBS2 knockdown, E-cadherin expressions were significantly elevated, N-cadherin, vimentin and Snail expressions were significantly diminished, indicating that the EMT process of BL cells was significantly inhibited.

The imbalance of cell cycle regulation is the core mechanism that drives the malignant proliferation of tumors (34, 35). The dysregulation of the cell cycle affects the cell cycle through a variety of ways, including regulating Cyclin and CDK, thereby inhibiting the proliferation of tumor cells in various types of malignant tumors. Yu et al. found that activation of the PI3K/AKT/c-MYC signaling pathway triggers cell cycle arrest, thereby delaying the growth of bladder cancer (25). Similarly, knockdown of THBS2 effectively inhibited CDK2, CDK4, CDK6 and Cyclin D1 proteins, and arrested cell cycle.

The core finding of this study is that THBS2 affects BL cell malignant progression by activating the PI3K/AKT pathway and regulating c-MYC expression. We observed that after THBS2 knockdown, the phosphorylation of PI3K and AKT was significantly decreased, and c-MYC expression was down-regulated. The PI3K agonist 740Y-P partially restored the PI3K/AKT/c-MYC signaling pathway, while c-MYC overexpression reversed the inhibition of proliferation, suppression of invasion and migration, and induction of apoptosis caused by THBS2 knockdown. This mechanism is highly consistent with the findings of several research teams in recent years. Yao et al.’s latest study showed that in prostate cancer, THBS2 stimulates the PI3K/AKT pathway, promotes tumor growth, and inhibits apoptosis (21). Another study showed that THBS2 + CAFs promoted EMT through COL8A1-mediated PI3K/AKT activation, leading to oxaliplatin resistance (36). Thus, the THBS2/PI3K/AKT axis is a conserved cancer-promoting regulatory pathway in various tumors. We further investigated the molecular mechanism by which THBS2 regulates c-MYC activity. As a BL driver gene, c-MYC functions by forming heterodimers with MAX, which then binds to E-box elements to activate target gene transcription (26, 37). We confirmed that after THBS2 knockdown, the enrichment level of c-MYC in the promoter regions of CAD, CDK4, LDHA, NCL, PKM2 and HES1 was significantly reduced; moreover, THBS2 deficiency attenuated the dimerization of MYC/MAX. This finding reveals the regulatory effects of THBS2 in c-MYC transcriptional activities and provides a new perspective for understanding the mechanism by which THBS2 promotes cancer.

Cancer stem cells possess the capability for infinite proliferation, self-renewal, and differentiation into multiple cell types (38). The existence of cancer stem cell subsets is considered to be a core driver of chemotherapy resistance and disease recurrence (39). SOX2 and OCT4 are cancer stem cell markers, which are involved in the malignant process (40). SOX2 and OCT4 expression is often increased in cancer cells (41). In addition, recent studies in gastric cancer have shown that THBS2 enhances tumor development and stemness maintenance, including SOX2 expression (42). This study also found that c-MYC is associated with regulating BL cell stem cell-like characteristics. Knockdown of c-MYC inhibited stem cell sphere-forming ability and down-regulated the expression of SOX2 and OCT4. This suggests that THBS2 may regulate tumor stemness through the PI3K/AKT/c-MYC axis, forming an intricate regulatory network.

Since *in vitro* experiments cannot fully evaluate the effectiveness and feasibility of THBS2 on BL intervention measures, the establishment of animal models has an irreplaceable role in simulating the pathological state of human BL and evaluating treatment measures. SCID mice are genetically engineered mice lacking T and B lymphocytes. Due to the lack of adaptive immune response, SCID mice have become indispensable in oncology research, particularly for constructing human tumor xenograft models (43). Subcutaneous inoculation is easy to dynamically observe the proliferation of tumors because of its superficial tumor location, and the experimental period is short, which has little effect on mice. This method can more intuitively observe the effect of THBS2 on BL animals *in vivo*. In this study, RAJI cells with stable THBS2 knockdown were inoculated into SCID mice. The cells were inoculated subcutaneously in mice, and the BL animal model was successfully constructed. Ki67 and PCNA are markers of cell proliferation, which can be expressed at various stages of cell proliferation, and their expression levels can reflect the proliferation ability of tumor cells (44, 45). Knockdown of THBS2 could effectively inhibit tumor growth, reduce Ki67 and PCNA proteins, and promote apoptosis. Moreover, PI3K agonist 740Y-P or MYC overexpression can partially reverse the tumor inhibition caused by THBS2 knockdown, providing direct functional evidence for the regulation of THBS2/PI3K/AKT/c-MYC signaling axis.

This study still has some limitations. Firstly, the expression of THBS2 in BL tissues and its relationship with clinicopathological features and prognosis need to be further verified. THBS2 is strongly associated with the poor prognosis of patients with gastric cancer and other solid tumors (46–48), but its clinical significance in BL still needs to be confirmed by large sample cohort studies. Secondly, the upstream mechanism of THBS2 regulating PI3K/AKT pathway is not fully understood. THBS2 can bind to receptors (CD36 and integrin) (18), or activate downstream signals in an INHBA-dependent manner (21). However, the specific receptors and initial signal events of THBS2 in BL cells still require further investigation.

## 5 Conclusion

This study confirmed that THBS2 acts as an oncogene in BL. Knockdown of THBS2 inhibits cell malignant phenotype, EMT and stem cell-like characteristics by suppressing the PI3K/AKT/c-MYC axis, while also promoting apoptosis and inducing cell cycle arrest. This research offers a novel perspective for understanding the molecular pathological mechanism of BL and provides an experimental basis for therapeutic strategies targeting the THBS2/PI3K/AKT/c-MYC pathway. Future studies can further explore the clinical value of THBS2 as a prognostic marker for BL, and the feasibility of developing small molecule inhibitors targeting THBS2 or its downstream pathways for BL treatment.

## Data Availability

The original contributions presented in the study are included in the article/**Supplementary Material**. Further inquiries can be directed to the corresponding author.
